# Was the COVID-19 epidemic synchronous in space? An analysis in the health regions of the Rio de Janeiro state, 2020-2022

**DOI:** 10.1590/1980-549720240010

**Published:** 2024-02-26

**Authors:** Léa de Freitas Amaral, Raquel Martins Lana, Leonardo Soares Bastos

**Affiliations:** I Fundação Oswaldo Cruz, National School of Public Health - Rio de Janeiro (RJ), Brazil.; II Barcelona Supercomputing Center - Barcelona, Spain.; III Fundação Oswaldo Cruz, Scientific Computing Program - Rio de Janeiro (RJ), Brazil.

**Keywords:** COVID-19, Spatio-temporal analysis, Hospitalization, Rio de Janeiro, COVID-19, Análise espaço-temporal, Hospitalização, Rio de Janeiro

## Abstract

**Objective:**

To analyze the spatio-temporal dynamics of COVID-19 in the Rio de Janeiro state within the nine health regions, between March 2020 and December 2022.

**Methods:**

The Poisson model with random effects was used to smooth and estimate the incidence of COVID-19 hospitalizations reported in the Influenza Epidemiological Surveillance Information System (SIVEP-Gripe) to verify the synchronicity of the epidemic in the state.

**Results:**

The COVID-19 epidemic in the state is characterized by the presence of seven peaks during the analyzed period corresponding to seven found. An asynchrony in hospitalizations was identified, varying according to the different virus variants in the nine health regions of the state. The incidence peaks of hospitalizations ranged from 1 to 12 cases per 100,000 inhabitants during the pandemic.

**Conclusion:**

This spatio-temporal analysis is applicable to other scenarios, enabling monitoring and decision-making for the control of epidemic diseases in different areas.

## RESUMO

**Objetivo:**

Analisar a dinâmica espaço-temporal de COVID-19 no estado do Rio de Janeiro nas nove regiões de saúde, entre março de 2020 e dezembro de 2022.

**Métodos:**

Utilizou-se o modelo de Poisson com efeitos aleatórios para suavizar a curva de incidência de hospitalizações por COVID-19 notificadas no Sistema de Informação da Vigilância Epidemiológica da Gripe (Sivep-Gripe) para verificar a sincronicidade da epidemia no estado.

**Resultados:**

A epidemia de COVID-19 no estado é caracterizada pela presença de sete picos no período analisado correspondentes a sete variantes encontradas. Identificou-se uma assincronicidade nas hospitalizações, variando de acordo com as diferentes variantes do vírus nas nove regiões de saúde do estado. Os picos de incidência das hospitalizações variaram de 1 a 12 casos por 100 mil habitantes no decorrer da pandemia.

**Conclusão:**

Essa análise espaço-temporal é extensível em outros cenários, sendo possível o monitoramento e a tomada de decisões de controle de doenças epidêmicas em várias áreas.

## INTRODUCTION

COVID-19 (coronavirus disease 2019) is an acute and potentially severe respiratory infection, of great importance for public health^1^, which has been declared by the World Health Organization (WHO) as a public health emergency of international concern (PHEIC) for three years^2^. COVID-19 is caused by the SARS-CoV-2 virus^1^ and may range according to severity, including asymptomatic cases and mild clinical manifestations, moderate cases in the form of pneumonia, as well as severe and critical cases, such as Severe A cute Respiratory Illness(SARI ), leading to the need for hospitalization^3^. This situation generated a high number of cases in a short space of time around the world, increasing the challenge for health systems^4^.

The SARS-CoV-2 virus has suffered mutations throughout the pandemic, which generated new variants, since it has extensively circulated in the population, causing many infections^5^. The first identified variants were Gama (P.1), in December, 2020, in Manaus, capital of Amazonas, and Zeta (P.2), identified in October, 2020, in the Rio de Janeiro state. Respectively, they were classified as variants of concern (VOC) and interest (VOI), according to the WHO classification^6^ ^,^ ^7^. The epidemic has spread fast and became worse with the disorganization of the health system, as well as the lack of efficient public policies for control^8^.

**Figure 1.**
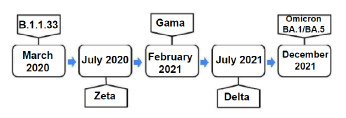
Time of entry of each one of the variants in the Rio de Janeiro state.

The Rio de Janeiro statewas one of the main viral dissemination poles for other Brazilian locations. During the initial phase of the pandemic in the country, SARS-CoV-2 first spread locally and within state borders. The motion of the virus lineage in the state was more frequent than that in other states^5^. Without a nationally coordinated strategy, states and cities varied in form, intensity and duration of implementation of pharmacological and non-pharmacological measures^9^. Figure 1 shows, chronologically, the moment when variants entered the state.

Considering the importance of knowing about the dynamics of disease transmission in one of the main transmission poles in the country, the objective of this study was to characterize the synchronicity of spatiotemporal dynamics of the COVID-19 epidemic in the Rio de Janeiro state . The nine health regions of the state were chosen as units of analysis from March 2020 to December 2022, using hospitalization data from the Influenza Epidemiological Surveillance Information System (Sivep-Gripe). The analysis provided relevant information about the occurrence of an epidemic in the state, with potential to be used in other disease situations involving epidemic characteristics. The presented results are useful as a surveillance protocol for future conditions. Therefore, the analysis is an important tool to characterize epidemics in different Brazilian regions and around the world, increasing the knowledge in public health and promoting the preparation for the emergence of new variants of SARS-CoV-2 , and emergence or re-emergence of other communicable diseases.

## METHODS

### Study area

**Figure 2.**
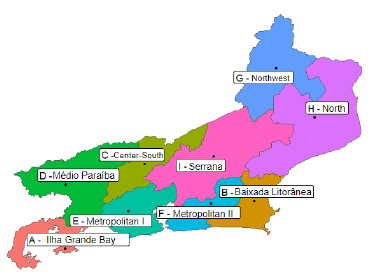
Map of the Rio de Janeiro state divided in nine health regions.

The study area contemplates the entire Rio de Janeiro state, which is located in the Southeast region, with an area of 43,781,588 km². The state has 92 cities distributed in nine health regions: Ilha Grande Bay, Baixada Litorânea, Center-South, Médio Paraíba, Metropolitan I, Metropolitan II, Northwest, North and Serrana (Figure 2).

### Data

The data regarding cases of patients hospitalized due to COVID-19 used in this study were obtained in secondary bases from the Ministry of Health, via Sivep-Gripe, which includes information about SARI. These data are available in OpenDataSUS (https://opendatasus.saude.gov.br/).

Data referring to population size of the health regions came from aggregations of population projection numbers per city, carried out by the Laboratory of Estimations and Population Projections (LEPP) in the Postgraduate program in Demography (PPGDem), of the Department of Demography and Actuarial Sciences (DDCA) of Universidade Federal do Rio Grande do Norte (UFRN). The projections are available at (https://demografiaufrn.net/projecao-populacional/).

### Case definition

A case of hospitalization due to SARI-COVID was defined by a hospitalized individual notified in Sivep-Gripe, who tested positive for SARS-CoV-2 in the RT-PCR molecular test and (or) reactive to the antigen test, and (or) presenting epidemiological clinical criteria.

### Data Analysis

To verify the synchronicity of the epidemic in the Rio de Janeiro state , we used time series of SARI-COVID cases in the nine health regions of the state, on the ninth epidemiological week of 2020 (from February 23 to 29, 2020), until the 52^nd^ epidemiological week of 2022 (from December 25 to 31, 2022). The expected incidence per week and health region, (λ ^(t,r)^), was estimated based on the following random effect Poisson model:

$$H^{\left(t,r\right)}\;\;\sim \;Poisson(\lambda^{\left(t,r\right)})$$

$$log\;(\lambda^{\left(t,r\right)})\;=\;{POP}^{\left(r\right)}\;exp\;(\beta^{\left(r\right)}\;+\;\theta^{\left(t,r\right)})$$

*t* =: 1, 2, …
T =: 159 weeks
*r* =: 1, 2, …, 9 health regions

In this formula, *H* ^(t,r)^ is the number of SARI-COVID cases on week *t*, and health region *r,* POP^(r)^, the population of region *r*, β^(r)^ is a fixed common effect (intercept) for regional *r*, and random effects θ^(t,r)^ follow the Gaussian model with time dependence of second order random walk, that is, the effect of week *t* on region *r* depends only on what happened in the two weeks before in that region, and assuming that all health regions are conditionally independent, that is, do not have a spatial structure.

The weekly expected incidence, (λ ^(t,r)^), was estimated for all health regions under the Bayesian approach, using the Inla method (Integrated Nested Laplace Approximations), which is a statistics technique used to allow the approximation of *a posteriori* distribution of complex hierarchical models in a fast and precise manner^10^.

Time was divided according to waves, in which each wave is defined by a continuous period of time, with the domain of one variant. One variant is described as dominant while being the most frequent one in sequencings deposited in a virus sequencing repository. The dates of beginning and end of each wave were defined based on sequencing data of the Rio de Janeiro state , compiled by the genomic network of Fundação Oswaldo Cruz (https://www.genomahcov.fiocruz.br/). Since we are evaluating the synchronicity of the epidemic, the division of time in waves is the same for all health regions, and we used data from the entire Rio de Janeiro state . The moment when the expected incidence reaches its highest value was calculated in each wave and region. This value is called peak. Synchronicity was assessed based on Metropolitan Region I (MRI) as reference, which contains the state capital. If the peak week of a specific region coincided with the weak peak of MRI, then, in this wave, the epidemics were considered as synchronous; otherwise, they were asynchronous. The analysis was carried out in R^11^.

## RESULTS

Until March 19, 2020, only 23 days after the first confirmed case in Brazil, the Rio de Janeiro state had already registered 64 confirmed cases, spread in five cities: Rio de Janeiro (55), Niterói (6), Barra Mansa (1), Miguel Pereira (1) and Guapimirim (1). Five of the nine health regions already identified the circulation of the virus: Metropolitan I, Metropolitan II, Médio Paraíba, Center-South and Serrana^12^. The COVID-19 epidemic in the state can be characterized with seven waves in the analyzed period. These waves correspond to the following variants: B.1.1.33, Zeta, Gama, Delta and Omicron (subvariants BA.1, BA.2 and BA.5).

**Figure 3.**
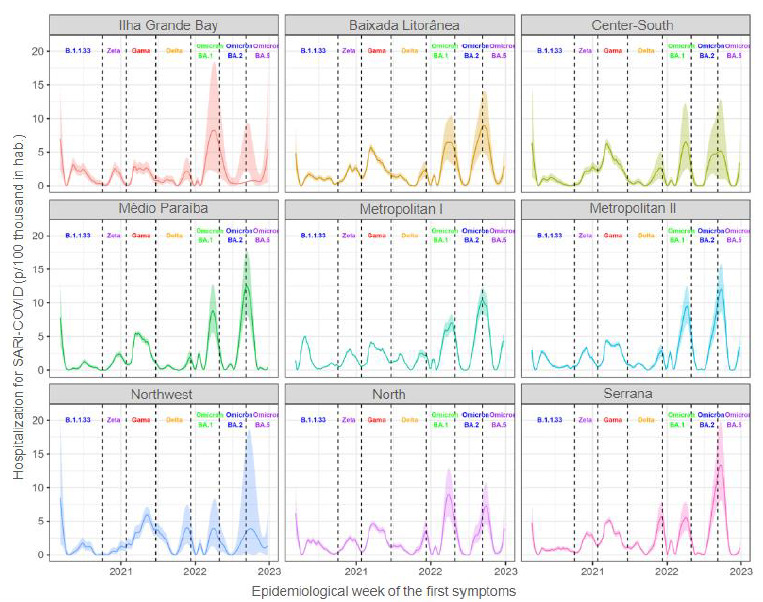
Expected incidence, with credibility interval of 95%, of hospitalizations due to COVID-19 in the nine health regions of the Rio de Janeiro state, between February 23, 2020 (EW 09) and December 31, 2022 (EW 52).

In Figure 3, it is observed that, in the first wave, the nine health regions presented different moments of increase in hospitalizations. The predominant variable in the state was B.1.1.33. Metropolitan Region I was the one with the highest incidence, approximately five cases per 100 thousand inhabitants. Variant B.1.133 did not present a well-defined peak in the following regions: Baixada Litorânea, Center-South and Serrana, where the incidence of hospitalizations remained high during the entire period; it was not possible to define a peak. In the second wave, corresponding to the Zeta variant, and in the third wave, corresponding to the Gama variant, the highest hospitalization rates were in the following regions: Baixada Litorânea, Center-South and Serrana. In both moments, incidence rates reached more than 2.5 cases per 100 thousand inhabitants in these regions. The Zeta variant did not present a well-defined peak only in the Northwest region, whereas the Gama variant had a defined peak in all nine regions of the state. In the fourth wave, the prevalent variant in the state was Delta, which did not present a well-defined peak in the regions of Ilha Grande Bay, Baixada Litorânea, Northwest and North. In this context, the Serrana region had higher incidence of hospitalizations, approximately five cases per 100 thousand inhabitants. The Omicron variant, which circulated in the state from December 2021 until the end of the study (December, 2022), is the only variant that presents three waves (subvariants BA.1, BA.2 and BA.5) in the nine regions; the ones with highest incidence rates were: Médio Paraíba, Metropolitan II and Serrana, ranging from 1 to 12 cases per 100 thousand inhabitants when Omicron was dominant in the state.

**Figure 4.**
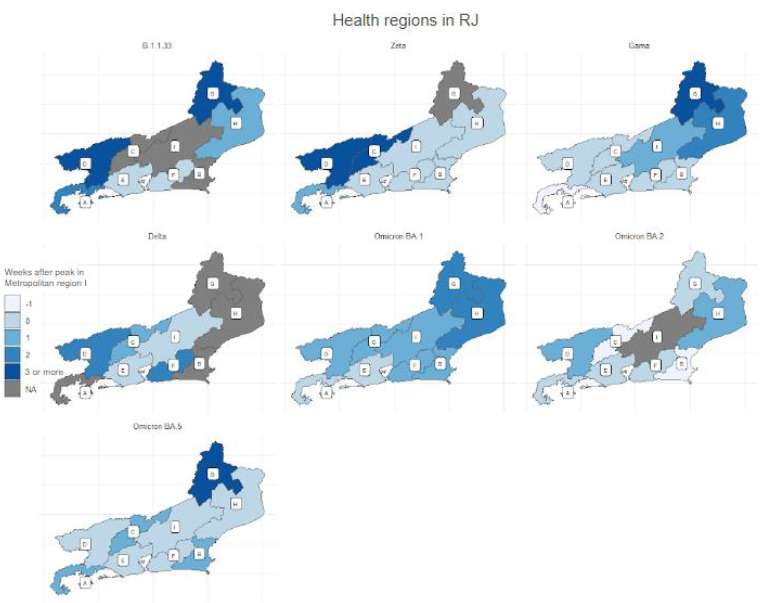
Map of health regions in the Rio de Janeiro state of all variants, identifying the weeks after the peak in Metropolitan region I.

Figure 4 shows the difference in time between the peak moment of each region and the peak moment of Metropolitan Region I (E). The regions in grey in the map represent places that did not have well-defined hospitalization waves. For variant B.1.1.33, this occurred in Baixada Litorânea, Center-South and Serrana. The regions in the extremes of the state, Northwest and Médio Paraíba were the ones that took three weeks or more to have their peaks, followed by the Ilha Grande Bay, in two weeks, and North, in one week. There was synchronicity between Metropolitan regions I and II.

A: Ilha Grande Bay; B: Baixada Litorânea; C: Center-South; D: Médio Paraíba; E: Metropolitan I; F: Metropolitan II; G: Northwest; H: North; I: Serrana; NA: it means that in that region and period/wave, there was no peak.

The Zeta variant was the one with most synchronicity, being observed in five regions: Metropolitan I and II, Baixada Litorânea, Serrana and North. This one did not present a defined peak in the Northwest region; it took one week to present its peak in Ilha Grande Bay and three weeks or more in Center-South and Médio Paraíba. For the Gama variant, the peak in the Ilha Grande Bay region occurred one week before it did in Metropolitan region I. There was synchronicity between five regions: Metropolitan I, Metropolitan II, Baixada Litorânea, Center-South and Médio Paraíba. In the Serrana region, it took one week to occur, whereas in the North region it took two weeks; in the Northwest region, it took three weeks or more.

The Delta variant only presented synchronicity between Metropolitan I and Serrana regions. The Center-South region had its peak after one week; Metropolitan II and Médio Paraíba, after two weeks; and Northwest, North, Baixada Litorânea and Ilha Grande Bay did not have a defined peak. The Omicron variant BA.1 was present in all regions; however, its peak took two weeks to occur in the Northwest and North regions; one week, in Metropolitan II, Baixada Litorânea, Serrana, Center-South and Médio Paraíba. The peak in Ilha Grande Bay was synchronous with Metropolitan I. The peak of Omicron BA.2 subvariant was synchronous in regions of Ilha Grande Bay, Northwest, Metropolitan I and II, but did not have a well-defined peak in the Serrana region, whereas it happened one week before in the Center-South and Baixada Litorânea. A late peak, of one week, was observed in Médio Paraíba and North regions. The Omicron BA.5 variant presented synchronicity between Center-South, Metropolitan I, Metropolitan II, Serrana and North regions. It took one week for the peak to occur in the Ilha Grande Bay, Médio Paraíba and Baixada Litorânea, and three weeks or more in the Northwest region.

## DISCUSSION

The main objective of this study was to carry out a spatiotemporal synchronicity analysis of the COVID-19 epidemic in the health regions of the Rio de Janeiro state , including the period from March 15, 2020 (EW 9) to December 31, 2022 (EW 52). The results of this spatiotemporal analysis revealed an introduction and asynchronous increase of confirmed cases of COVID-19 in the different health regions of the Rio de Janeiro state , showing seven waves throughout the study period.

The results pointed out to a first peak related to the B.1.1.33 variant, with the highest incidence of hospitalizations concentrated in Metropolitan Region I, which is the area that contains the state capital, the city of Rio de Janeiro. The fact that the city of Rio de Janeiro is an important entry point for national and international travelers, besides being a globally known tourism destination, may have contributed with the early introduction of the virus in the territory, making it the second city in the country to register confirmed cases of COVID-19^13^.

The synchronicity analysis showed that, after the B.1.1.33 variant, there was a significant increase in cases not only in Metropolitan region I, but also in all other health regions. Initially, the propagation of the virus was influenced by the existing socioeconomic inequalities between the regions, and the lack of a coordinated, efficient and equalitarian response favored the asynchronous and generalized dissemination of the virus^14^. The vulnerable population, who depends on public transportation and on the public health system, had more difficulties to adjust to social distancing measures due to poor household conditions and overcrowded vehicles, health units and hospitals^15^ ^,^ ^16^.

The dissemination of the subsequent variant, Zeta, showed a pattern of higher incidence of hospitalizations in the following regions: Serrana, Center-South, Metropolitan II and Ilha Grande Bay, indicating an expansion of the virus to the countryside of the state, with higher peaks than in Metropolitan Region I. Besides, there was a more frequent movement of the virus lineage within the state in comparison with the movement between states, reinforcing the importance of control measures to prevent outbreaks^13^. In that moment, control measures for the circulation of the virus had been reduced, which coincided with a second wave that began in November, 2020, in the electoral period of the country^17^. The cities softened restrictive measures in different moments, which facilitated the circulation of the virus in an asynchronous manner due to the mobility of the population, acting as a trigger for disease propagation^18^.

The introduction of the vaccination campaign against COVID-19, in January 2021, with the Gama variant in the state, played a significant role in the reduction of hospitalizations, but the highest incidence rates of hospitalization remained in the countryside regions, due to the non-homogeneous distribution of vaccines among cities. However, the incidence of hospitalizations after this variant decreased due to the ongoing vaccination campaign, which reduced the number of hospitalized patients, relieving the load on health systems and allowing the flexibilization of social distancing measures^19^ ^,^ ^20^.

The fourth identified variant, Delta, presented lower hospitalization rates than the previous variants, since vaccination had begun in the previous months. The CoronaVac vaccine, the first one to be available in Brazil, especially in the first days of vaccination, proved to be relatively efficient against the most severe cases of this variant and its predecessors, reducing cases of hospitalization^21^.

With the circulation of Omicron subvariants (BA.1, BA.2 e BA.5), there was an increase in cases in all health regions, even though the percentage of people who needed specialized care, such as hospitalization, was lower than in comparison to other variants. This variant presents several mutations with potential to increase transmissibility, providing resistance to therapy or partially escaping the immunity induced by infection or vaccine^22^. Mass vaccination stood out as an essential measure to prevent clinical complications caused by COVID-19. It is important to emphasize that this disease can be prevented from the point of view of clinical complications with high vaccine coverage^23^.

This study has limitations, even though hospitalizations are a fraction of cases, they are influenced by the vaccines. So, the use of hospitalizations to describe the dynamics of the epidemic in cases of COVID-19 in the Rio de Janeiro state is a study limitation. However, the data about hospitalizations are more robust to describe the dynamics of the COVID-19 epidemic than the data regarding cases, which face several biases, such as lack of an organized data system, unavailability of tests, lack of notification in self-test results, delay in notifications and quality of the filled out information.

The search for equality in vaccination in all cities of the state is recommended, aiming at the fast dissemination of the virus, preventing severe cases, preventing the appearance of new variants and reducing the demand for specialized health care. The knowledge of prevalent variables in each region allows for better planning of vaccine distribution and strategies of immunization, in order to increase vaccine coverage and reduce the dissemination of the virus. An additional and very relevant aspect is found in the supervision of the possible new ability of new variants to escape the immune response offered by the vaccines.

The results of this study have significant implications for the continuous monitoring of COVID-19 and the public health surveillance measures. When understanding the spatial and temporal dynamics of the epidemic, health administrators may address the available resources in a more efficient way, besides implementing preventive measures and punctual interventions in the most affected areas. Through this context, when implementing such monitoring in regions where the variant was previously established, and by identifying any signs of this occurrence, the warnings addressed to these areas where virus circulation is still less disseminated can be anticipated. The inclusion of these elements, when conducted in a concrete manner for surveillance, has the potential of considerably increasing a prompt response when facing this situation. This analysis can be applied in other epidemiological scenarios, allowing the monitoring of diseases with epidemic potential in different places, becoming an important tool to improve public health response, anticipating outbreaks and making decisions that are more based on the control of infectious diseases, thus contributing with the promotion of health and well-being of the population.
